# Device-based approaches for renal nerve ablation for hypertension and beyond

**DOI:** 10.3389/fphys.2015.00193

**Published:** 2015-07-08

**Authors:** Alicia A. Thorp, Markus P. Schlaich

**Affiliations:** ^1^Neurovascular Hypertension and Kidney Disease Laboratory, Baker IDI Heart and Diabetes InstituteMelbourne, VIC, Australia; ^2^School of Public Health and Preventive Medicine, Monash UniversityMelbourne, VIC, Australia; ^3^Department of Cardiovascular Medicine, Alfred HospitalMelbourne, VIC, Australia; ^4^Faculty of Medicine, Nursing and Health Sciences, Monash UniversityMelbourne, VIC, Australia; ^5^Royal Perth Hospital Unit, School of Medicine and Pharmacology, University of Western AustraliaPerth, WA, Australia

**Keywords:** sympathetic overactivity, renal denervation, blood pressure, resistant hypertension, cardiovascular disease, renal nerve activity

## Abstract

Animal and human studies have demonstrated that chronic activation of renal sympathetic nerves is critical in the pathogenesis and perpetuation of treatment-resistant hypertension. Bilateral renal denervation has emerged as a safe and effective, non-pharmacological treatment for resistant hypertension that involves the selective ablation of efferent and afferent renal nerves to lower blood pressure. However, the most recent and largest randomized controlled trial failed to confirm the primacy of renal denervation over a sham procedure, prompting widespread re-evaluation of the therapy's efficacy. Disrupting renal afferent sympathetic signaling to the hypothalamus with renal denervation lowers central sympathetic tone, which has the potential to confer additional clinical benefits beyond blood pressure control. Specifically, there has been substantial interest in the use of renal denervation as either a primary or adjunct therapy in pathological conditions characterized by central sympathetic overactivity such as renal disease, heart failure and metabolic-associated disorders. Recent findings from pre-clinical and proof-of-concept studies appear promising with renal denervation shown to confer cardiovascular and metabolic benefits, largely independent of changes in blood pressure. This review explores the pathological rationale for targeting sympathetic renal nerves for blood pressure control. Latest developments in renal nerve ablation modalities designed to improve procedural success are discussed along with prospective findings on the efficacy of renal denervation to lower blood pressure in treatment-resistant hypertensive patients. Preliminary evidence in support of renal denervation as a possible therapeutic option in disease states characterized by central sympathetic overactivity is also presented.

## Introduction

Treatment-resistant hypertension (rHTN) is a clinically important condition that is associated with increased cardiovascular morbidity and mortality risk (Daugherty et al., 2012; Irvin et al., 2014). Defined by the failure to achieve target blood pressure (BP) despite taking ≥3 antihypertensive medications at optimal doses, rHTN is estimated to affect 8−10% of all hypertensive adults (Persell, 2011; De La Sierra et al., 2011). Poor adherence to pharmacotherapy is ubiquitous in this patient cohort hindering efforts for timely and consistent BP management (Jung et al., 2013), which has caused some clinicians to question whether rHTN really exists or is a case of difficult-to-treat hypertension. Despite the controversy, there is compelling evidence that chronic sympathetic outflow to and from the kidneys is involved in the pathophysiology of rHTN (Esler et al., 1989; Schlaich et al., 2004; Smith et al., 2004).

The emergence of catheter-based renal denervation (RDN)- a minimally invasive procedure used to ablate renal sympathetic nerves- has marked a paradigm shift in the therapeutic management of rHTN. The long-term safety and efficacy of RDN to control BP has principally been evidenced by open-label studies. In the last 12-months, several rigorously designed, randomized controlled trials have brought into question the efficacy of RDN to treat rHTN. Specifically, data from the Symplicity HTN-3 study failed to show the primacy of RDN in lowering BP compared to a sham-procedure in rHTN patients (Bhatt et al., 2014).

In addition to rHTN, sympathetic overactivity is a cardinal feature of several pathological conditions including renal disease, heart failure, left-ventricular hypertrophy, insulin resistance, and sleep apnea. The ability of RDN to alter renal afferent signaling and reduce whole body sympathetic nerve activity in these disease states is currently being explored in a number of preclinical and clinical studies with promising results.

This review will we focus on the importance of renal sympathetic nerves in the pathophysiology of rHTN, technological advancements in ablation modalities for RDN and latest prospective clinical findings. Novel therapeutic applications for RDN beyond BP control will also be discussed along with several critical issues that must be addressed for research going forward.

## Renal nerves: an important target for blood pressure control

The kidneys are connected to the autonomic nervous system via a dense, neuronal network of post-ganglionic sympathetic nerve fibers located within the adventitia of the renal artery (Dibona, 2005). Renal efferent motor fibers innervate all parts of the renal vasculature, tubules, and juxtaglomerular apparatus (Barajas, 1978); while afferent sensory nerves are located principally in the renal pelvic wall and serve to connect the kidneys with autonomic centers in the central nervous system (Kopp, 1992).

**Figure 1:**
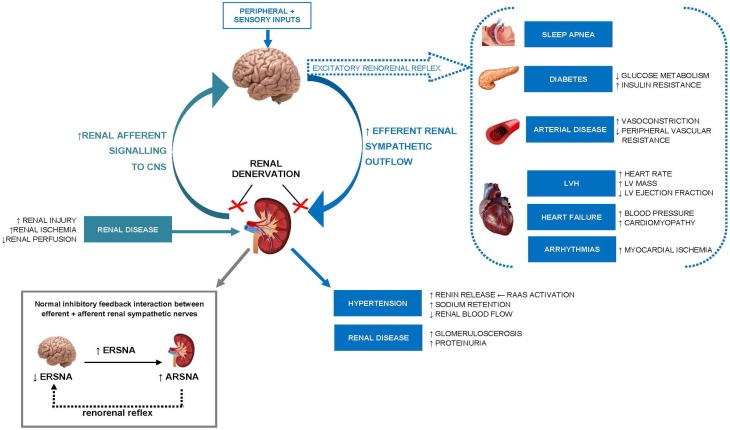
**Schematic illustration of the role of increased renal efferent sympathetic outflow and increased renal afferent sensory signaling in the pathophysiology of hypertension and other cardiovascular, renal, and metabolic disease states**. ERSNA, efferent renal sympathetic nerve activity; ARSNA, afferent renal sympathetic nerve activity; RAAS, renin–angiotensin–aldosterone system; LVH, left ventricular hypertrophy; LV, left ventricular; CNS, central nervous system.

Animal (Dibona and Kopp, 1997) and human (Esler, 2000) studies suggest chronic activation of renal sympathetic nerves is important in the pathogenesis and perpetuation of essential hypertension as well as other pathological conditions including heart failure, chronic kidney disease, and diabetes (see review Malpas, 2010). As highlighted in Figure 1, sustained efferent sympathetic outflow to the kidneys (via peripheral and central sensory inputs) elevates BP by altering renal vascular resistance (Kirchheim et al., 1989), stimulating renin release from juxaglomerular granular cells (Zanchetti, 1977) and increasing tubular sodium and water reabsorption (Bell-Reuss et al., 1976). Excitatory reflexes originating from afferent renal nerves in the kidney can also contribute to development of hypertension, particularly rHTN (Hering et al., 2014). Under normal physiological conditions, changes in hydrostatic pressures or chemical composition of the renal environment activate sensory afferent renal nerves to stimulate a centrally-mediated decrease in efferent renal sympathetic outflow via an inhibitory feedback mechanism, known as the renorenal reflex response.

Pathological activation of renal sensory afferent nerve fibers through renal ischemia, injury or elevated adenosine concentrations (Esler, 2010), can alter the activity of central integrative neuronal circuits involved in cardiovascular homeostasis and shift the renorenal reflex response from inhibitory to excitatory. The consequence is an increase in central efferent sympathetic outflow to the kidneys and to other highly innervated organs (such as the heart and vasculature) initiating the development and/or maintenance of hypertension and other pathological conditions.

The application of radiofrequency energy to ablate renal nerves in the adventitia can reduce efferent renal sympathetic activity [as evidenced by a reduction in noradrenaline spillover in the kidney (Krum et al., 2009)] to promote urinary sodium excretion and BP reduction; while the ablation of afferent renal sympathetic nerves lowers BP via the inhibition of central sympathetic outflow (Schlaich et al., 2009a).

## Changing face of renal denervation: latest developments in device-based nerve ablation

Concomitant with the refinement of radiofrequency-based catheter ablation systems, there has been an emergence of novel treatment modalities that use highly differentiated approaches (i.e., ultrasonic nerve, pharmacological, and cryoablation) to ablate renal sympathetic nerves. These next-generation RDN systems may potentially offer several clinical advantages to radiofrequency-based modalities namely the ability to deliver energy circumferentially and penetrate deeper into the adventitia to optimize neural damage, while preserving artery integrity.

Intravascular and fully non-invasive ultrasound nerve ablation modalities have undergone extensive clinical investigation in the last 12–18 months (refer Table 1). Intravascular ultrasound ablation modalities use transducer-based catheters to deliver high-intensity, non-focused ultrasonic emissions (rapid mechanical oscillations) to renal nerves at a depth range of 0.5–10 mm. The latest iteration of the ReCor Percutaneous Renal Denervation System (the RADIANCE™ catheter) uses a novel radial access approach to deliver ultrasonic waves to the renal adventitia, promoting minimal invasiveness to the patient. The therapeutic intra-vascular ultrasound (TIVUS™) catheter system (Cardiosonic Ltd, Tel Aviv, Israel) similarly delivers ultrasonic waves to the renal adventitia but in the absence of endoluminal surface contact, preserving the integrity of the artery. Results from first-in-man studies highlight the efficacy and safety of these latest modalities to reduce BP short-term (Jonas et al., 2014; Shetty et al., 2014; Daemen and Van Mieghem, 2015) with prospective, multi-center, clinical studies currently underway.

**Table 1:** **Overview of emerging intravascular and non-invasive ultrasound modalities for circumferential renal nerve ablation**.

**Product name**	**RDN modality**	**Description/feature**	**CE mark**	**Clinical trial data**	**Ongoing/planned trials**
ReCor RADIANCE catheter system	Intravascular ultrasound nerve ablation	Cylindrical catheter advanced into renal artery via radial access. 3 unfocused, ultrasounic emissions delivered bilaterally.	Yes	First-in-man study (*n* = 2) At 3-mo follow-up mean decline in office BP (−40/−29 mmHg) and 24-h ambulatory BP (−11/−8 mmHg).	Prospective, single-arm, open-label study (REALISE Trial; *n* = 20)- ongoing not recruiting patients.
Cardiosonic TIVIS catheter system	Intravascular ultrasound nerve ablation	Catheter delivers high-frequency, high-intensity directional ultrasound emissions. Radiopaque tip positions catheter using fluoroscopic guidance. No endoluminal surface contact is required.	Yes	First-in-man study (TIVUS I; *n* = 18 with rHTN). Prospective, non-randomized, single-arm, open label study. Mean decline in office BP at 1-mo (−28/−10 mmHg; *n* = 18) and 3-mo follow-up (−25/−10 mmHg; *n* = 16).	Prospective, multicenter, non-randomized, single-arm, open-label clinical study using system next generation Multidirectional Catheter TIVUS™ (TIVUS II). Study will include treatment arm for patients who have failed radio-frequency RDN. To date 6 patients have been enrolled in TIVUS™ II – ongoing and recruiting patients.
Sound Interventions SOUND 360 catheter system	Intravascular ultrasound nerve ablation	Cylindrical transducer encased in a non-cylindrical, non-occlusive balloon delivers therapeutic ultrasound at specific dosimetry. 2 unfocused ultrasonic emissions delivered bilaterally.	No	First-in-man study (SOUND-ITV; *n* = 10 with rHTN). Mean decline in office BP (−25.6/−12.5 mmHg) and 24-h ambulatory BP (−23.1/−11.9 mmHg) at 3-mo follow-up. 3 patients developed groin hematomas not requiring intervention.	Nil
Kona Medical Surround Sound Hypertension Therapy system	Non-invasive ultrasound nerve ablation	Externally focused, low frequency ultrasonic emissions delivered bilaterally to the renal adventitia.	No	First-in-man study (WAVE I; *n* = 24) At 6-mo follow-up, mean −27 mmHg decline in office SBP. WAVE II trial: *n* = 13 with rHTN. At 6-week follow-up (*n* = 8), mean −18 mmHg decline in office SBP, no decline in office DBP. No serious adverse events. WAVE III trial- completed, results not yet disclosed.	Multi-center, randomized, sham-controlled, double-blind trial (WAVE IV; target *n* = 132). Study will include treatment arm for patients who have failed alternate forms of RDN-ongoing and recruiting patients.

*rHTN, resistant hypertension*.

The Surround Sound Hypertension Therapy System (Kona Medical, Washington, USA) is the only fully external ultrasound-based RDN system being investigated as an adjunct therapy to medication in rHTN patients. Circumferential nerve ablation is achieved using externally focused, low-frequency ultrasonic waves delivered to the renal adventitia via an ultrasound imaging transducer. Initial data from a series of small clinical trials (WAVE I and WAVE II) found that 81% of patients (total *n* = 41 across the two studies) experienced clinically significant reductions in office SBP (≥10 mmHg) at 6-months follow-up with no serious adverse events reported (Neuzil et al., 2014; Ormiston et al., 2014). Primary outcomes from a multi-center randomized, sham-controlled, double-blinded trial involving 132 subjects (WAVE IV) are expected to be released later this year. The findings will be of interest as only the second, sham-controlled clinical trial in rHTN patients and first to include a treatment arm of patients who have failed radiofrequency RDN modalities (Daemen and Van Mieghem, 2015).

## Clinical findings update: where is the evidence-base on renal denervation?

In the past 12-months there have been 5 prospective, randomized controlled trials that have reported either a modest or no effect of RDN on BP reduction in patients with rHTN (Bhatt et al., 2014; Fadl Elmula et al., 2014; Azizi et al., 2015; Desch et al., 2015; Rosa et al., 2015). Of most relevance is the Symplicity HTN-3 study, the largest and most rigorously designed trial to date, which failed to meet its primary efficacy endpoint (mean 6-month change in office SBP) (Bhatt et al., 2014). Previously, the only prospective trial to compare the BP lowering effects of RDN to usual care had been the open-label Symplicity HTN-2 Study (Esler et al., 2010), which reported a significant office BP reduction of −32/−12 mmHg at 6-months following RDN. The Symplicity HTN-2 study is recognized as having several limitations, the most notable being the use of office BP over 24-h ambulatory BP monitoring to assess the efficacy of RDN and the absence of a blinded control drug adherence monitoring in the study.

As the first randomized, double-blinded, sham-controlled trial, the Symplicity HTN-3 study was expected to provide the definitive statement on the superiority of RDN in the treatment of severe rHTN (Bhatt et al., 2014). A total of 535 patients were assigned in a 2:1 ratio to receive either the RDN or sham-procedure. Treatment rHTN was confirmed at baseline using 24-h ambulatory BP monitoring following 2-weeks of stable, maximally tolerated doses of ≥3 antihypertensive medications of complementary classes (including a diuretic). The 6-month follow-up data revealed significant office BP reductions in both treatment groups (RDN: −14.1/6.8 mmHg vs. SHAM: −11.7/4.8 mmHg; both *p* < 0.001). However, between-treatment differences in office BP reduction at 6-months were not significant (−2.4/2.0 mmHg; *P* = 0.26). Concomitantly, no superior treatment effect of RDN over the sham-procedure for mean change in 24-h (*p* = 0.98), daytime (*p* = 0.52), or night-time (*p* = 0.06) ambulatory SBP was observed (Bakris et al., 2014).

Clearly, the failure of Symplicity HTN-3 to show a clear-cut superiority of RDN over the sham-procedure in lowering BP (modest ~2 mmHg SBP reduction only) was disappointing, but contemplated by some (Howard et al., 2013). Evidence from a recent meta-analysis that combined office and ambulatory BP data from 10 European centers predicted similar, modest 6-month BP reductions following RDN and large variability in patient's BP responsiveness (Persu et al., 2014). A number caveats with the Symplicity HTN-3 study design have since been identified that may well account for some of the neutral findings. Specifically, despite the inclusion of a sham-procedure, the majority of interventional cardiologists were inexperienced in performing the procedure (one third performed one procedure only), only 6% of patients received bilateral circumferential ablation (as per protocol) with energy preferentially applied to the proximal portion of the renal artery, renal and total body noradrenaline spillover testing (measure of renal efferent activity and central sympathetic drive via renal afferent pathway respectively) was not performed, and patient's medication regime prior to testing at baseline and 6-months was not stable.

In the aftermath of the Symplicity HTN-3 study announcement, 3 rigorously designed, prospective, open-label randomized controlled trials using the same single-electrode Symplicity radiofrequency catheter system (Medtronic Inc, Minnesota, USA) have been published (Fadl Elmula et al., 2014; Azizi et al., 2015; Rosa et al., 2015). As highlighted in Table 2, two of the studies showed intensive pharmacotherapy to be equally effective (Fadl Elmula et al., 2014; Rosa et al., 2015) to RDN in lowering BP in patients with true rHTN, highlighting the ability of RDN to lower BP at least to the extent of additional pharmacologic treatment. The third study (DENER-HTN) comparing RDN in combination with standardized, stepped-care antihypertensive treatment (SSAHT) observed a modest, albeit significant reduction in 6-month daytime SBP [adjusted mean difference of −5.9 mmHg (95%CI: −11.3, −0.5); *p* = 0.03] compared to SSAHT alone (Azizi et al., 2015).

**Table 2:** **Randomised controlled trials using renal nerve ablation in the treatment of resistant hypertension in the last 12-months**.

**Study ID**	**Symplicity HNT-3 (Bhatt and Bakris, 2014)**	**PRAGUE-15 study (Rosa et al., 2015)**	**DENER-HTN trial (Azizi et al., 2015)**	**Symplicity FLEX study (Desch et al., 2015)**	**OSLO RDN study (Fadl Elmula et al., 2014)**
Study design	Prospective, single-blind multi-center RCT with 2:1 treatment (RDN v SHAM) randomization	Prospective, open-label, multi-center RCT with 1:1 treatment randomization	Prospective, single-blind, multi-center RCT with 1:1 treatment randomization	Prospective, open-label RCT with 1:1 treatment randomization	Prospective, open-label RCT with 1:1 treatment randomization
Control	Sham-procedure (renal angiography)	Intensive pharmacotherapy with spironolactone	Standardized stepped-care antihypertensive treatment (SSAHT)	Sham-procedure (renal angiography)	Antihypertensive drug adjustment
Patient Cohort	RDN: *n* = 364 Mean age: 58 ± 10 years Number of BP medications: 5.1 ± 1.4 SHAM: n=171 Mean age: 56 ± 11 years Number of BP medications: 5.2 ± 1.4	RDN: *n* = 52 Mean age: 56 ± 12 years Mean office SBP:159 ± 19 mmHg Number of BP medications: 5.1 ± 1.2 PHAR: n=54 Mean age: 59 ± 9 years Mean office SBP:155 ± 17 mmHg Number of BP medications: 5.4 ± 1.2	RDN + SSAHT: *n* = 53 Mean age: 55 ± 11 years Mean office SBP:159 ± 23 mmHg SSAHT: n=53 Mean age: 55 ± 10 years Mean office SBP:156 ± 22 mmHg	RDN: *n* = 35 Mean age: 65 ± 8 years Mean daytime SBP:144 ± 5 mmHg Number of BP medications: 4.4 ± 1.3 SHAM: n=36 Mean age: 57 ± 9 years Mean daytime SBP:143 ± 5 mmHg Number of BP medications: 4.3 ± 1.3	RDN: *n* = 9 Mean age: 57 ± 11 years Mean office SBP:156 ± 13 mmHg Number of BP medications: 5.1 ± 1.6 PHAR: n=10 Mean age: 63 ± 5 years Mean office SBP:160 ± 12 mmHg Number of BP medications: 5.0 ± 1.2
RDN Modality	Symplicity single-electrode radiofrequency catheter system (Medtronic Inc.)	Symplicity single-electrode radiofrequency catheter system (Medtronic Inc.)	Symplicity single-electrode radiofrequency catheter system (Medtronic Inc.)	Symplicity FLEX multi-electrode radiofrequency catheter system (Medtronic Inc.)	Symplicity single-electrode radiofrequency catheter system (Medtronic Inc.)
Drug Adherence	Patient diary	Plasma drug concentration	8-item Morisky Medication Adherence Scale	Patient diary	Witnessed intake
Primary Outcome	Change in 6-mo office SBP	Changes in 6-mo 24-h ambulatory BP	Change in 6-mo daytime ambulatory SBP	Change in 6-mo 24-h ambulatory SBP (intention-to-treat)	Change in 6-mo office SBP
Secondary Outcomes	Change in 6-mo 24-h ambulatory SBP	1-year spironolactone (PHAR) or medical (RDN) effects	Change in 6-mo ambulatory, home and office BP measures; eGFR; incidence of adverse events;	Change in 6-mo 24-h ambulatory DBP and mean ambulatory BP (intention-to-treat); change in 24-h ambulatory SBP (per-protocol)	Change in 3-mo and 6-mo daytime ambulatory BP
Results	Mean change in 6-mo office SBP RDN: −14.13 ± 23.93 mmHg SHAM: −11.74 ± 25.94 mmHg (both *P* < 0.001) No treatment differences for change in 6-mo office SBP (*p* = 0.26) or 24-h ambulatory SBP (*p* = 0.98)	Mean change in 6-mo 24-h ambulatory SBP: RDN: −8.6 mmHg (−11.8, −5.3) PHAR: −8.1 mmHg (−12.7, −3.4) (both *P* = 0.001) No treatment differences for change in 6-mo 24-h, daytime or night-time ambulatory BP (all *P* > 0.36)	Mean change in 6-mo daytime ambulatory SBP: RDN + SSAHT: −15.8 mmHg (−19.7, −11.9) SSAHT: −9.9 mmHg (−13.6, −6.2) Significant treatment difference for change in 6-mo daytime SBP (*p* = 0.03) in favor of RDN group	Mean change in 6-mo 24-h ambulatory SBP: RDN: −7.0 mmHg (−10.8, −3.2) SHAM: −3.5 mmHg (−6.7, −0.2) No treatment difference for change in 6-mo 24-h ambulatory SBP (*p* = 0.15) Per-protocol: significant treatment difference for change in 6-mo 24-h ambulatory SBP (*p* = 0.042) in favor of RDN group	Office SBP at baseline and 6-mo: RDN: 156 ± 13 vs. 148 ± 7 mmHg (*p* = 0.42) SHAM: 160 ± 14 vs. 132 ± 10 mmHg (*P* < 0.0005) Significant treatment differences for change in 6-mo office SBP (*p* = 0.002) and office DBP (*p* = 0.004) in favor of PHAR group
Conclusion	BP lowering effects of RDN is comparable to a sham-operation rHTN in patients with.	BP lowering effects of RDN is comparable to intensive pharmacotherapy in patients with true rHTN.	BP lowering effects of RDN + SSAHT is superior to SSAHT alone in patients with rHTN.	BP lowering effects of RDN is comparable to a sham-operation in patients with rHTN.	BP lowering effects of intensified pharmacological therapy is superior to RDN in patients with true rHTN.

*Data presented as mean ± SD or mean [95%CI]. RCT, randomized controlled trial; RDN, renal denervation; SHAM = invasive sham-procedure; PHAR, pharmacological treatment; rHTN, resistant hypertension; SBP, systolic blood pressure; DBP, diastolic blood pressure; mo, month*.

Despite their smaller cohort sizes, a major strength of these latest studies was the careful selection of patients with true treatment rHTN. Patients were only recruited if they had elevated ambulatory daytime systolic BP after witnessed intake (Fadl Elmula et al., 2014) or quantitative plasma/urine levels (Azizi et al., 2015; Rosa et al., 2015) confirmation of prescribed antihypertensive medications. The absence of a sham-procedure in favor of standardized pharmacological intervention in the studies was also compensated by the use of ambulatory BP monitoring to assess the efficacy of RDN (which is preferential to the use of office BP) in combination with drug adherence monitoring. Such measures help to minimize the potential over-inflation of treatment-effects caused by white coat hypertension or the Hawthorn effect, which is a criticism of the Symplicity HTN-3 study.

Preliminary data from a recent study using the Symplicity-FLEX catheter is somewhat more encouraging for RDN (Desch et al., 2015). The smaller study (*n* = 71) designed to emulate the Symplicity HTN-3 in patients with mild refractory hypertension (defined as daytime systolic BP of 135−149 mmHg and/or diastolic BP of 90−94 mmHg on ≥3 antihypertensive medications) found that in the per-protocol analyses, those who underwent RDN (*n* = 29) experienced a significant reduction in mean 24-h and daytime systolic BP at 6-months follow-up compared to patients treated with a sham-procedure (*n* = 34). On average, RDN reduced 24-h and daytime systolic BP by −4.8 mmHg (mean ± SD: −8.3 ± 8.9 mmHg vs. −3.5 ± 9.5 mmHg; *p* = 0.04) and −6.2 mmHg (mean ± SD: −9.9 ± 9.0 mmHg vs. −3.7 ± 9.9 mmHg; *p* = 0.01), respectively. In the intention-to-treat analyses (primary outcome) the significant treatment-effects observed in the per-protocol analyses group were attenuated (*p* = 0.15). The inclusion of patients who should a priori not have been eligible for the study has been suggested as a possible explanation (Desch et al., 2015).

## Beyond blood pressure control: other therapeutic uses of renal denervation

The localized effect of disrupting renal afferent signaling suggests RDN may offer distinct clinical benefits beyond BP control in pathological conditions characterized by central sympathetic overactivity. Specifically, there has been intense interest in the application of RDN in patients with renal disease, heart failure, and metabolic disorders (Mahfoud et al., 2011).

### Chronic kidney disease/dialysis

Excessive sympathetic nerve activity is a hallmark of both chronic kidney disease (CKD) and end-stage renal disease (ESRD). In CKD patients, augmented sympathetic tone is present in the early clinical stages of the disease and increases with disease progression (Grassi et al., 2011). Animal (Campese et al., 1995) and human (Converse et al., 1992; Grassi et al., 2011) studies have identified afferent signaling from diseased kidneys as playing a critical role in the progression of renal function decline. Indeed, in hypertensive patients, increased muscle sympathetic nerve activity is strongly associated with a decline in estimated glomerular filtration rate (eGFR) (Grassi et al., 2011). Bilateral nephrectomy is also shown to normalize elevated muscle sympathetic nerve activity in non-dialysis ESRD patients (Converse et al., 1992).

Despite the known importance of BP control for optimal renal function (Bakris et al., 2000) and mounting evidence that RDN does not induce structural or functional renal damage in hypertensive patients (Mahfoud et al., 2012; Dorr et al., 2013), international consensus states the procedure remain limited to patients with preserved renal indices. Hering et al. first reported on the efficacy and short-term safety of RDN in 15 moderate-to-severe CKD (mean creatinine-based eGFR 31.2 mL/min/1.73 m^2^) patients with rHTN (Hering et al., 2012). The study showed RDN safely reduced peripheral arterial stiffness and office BP while preserving renal blood flow, electrolytes and eGFR at 6-months follow-up. Pilot data from 24 predominantly stage 2 CKD patients (mean eGRF 64.4 mL/min/1.73 m^2^) with treatment rHTN (Kiuchi et al., 2013) showed a similar beneficial effect on 24-h ambulatory BP (mean reduction from baseline −19/−7 mmHg; *p* < 0.001) that was accompanied by short-term improvements in renal function. Compared to baseline, patients reported significantly higher eGFR and reduced urine albumin: creatinine ratio and serum creatinine (all *P* < 0.001) at 6-months follow-up. The conflicting short-term effects on eGRF reported between these two aforementioned pilot studies, suggests RDN may afford the greatest clinical benefit to patients in the early stages of CKD who have not yet undergone extensive vascular remodeling.

Two recent prospective longitudinal studies have reported sustained benefits in renal function parameters following RDN (Kiuchi et al., 2014; Ott et al., 2015). Kiuchi et al. observed of 27 CKD patients with rHTN that underwent RDN, those (*n* = 22) who achieved BP control (defined as office SBP <140 mmHg) at 12-months follow-up also reported a significantly higher eGRF (mean ± SD difference 18.54 ± 8.15 ml/min/1.73 m^2^; *p* = 0.03). Furthermore, improvements in office diastolic BP, serum creatinine and ACR were only observed at 12-months follow-up in patients who achieved BP control (*P* < 0.05 for all) (Kiuchi et al., 2014).

In a separate multi-center observational study (Ott et al., 2015), RDN was prospectively shown to reduce mean 24-h ambulatory BP (mean ± SD; 9 ± 14/4 ± 7 mmHg; *P* < 0.02) and improve mean eGFR by 1.5 ± 10 ml/min/1.73 m^2^ (*p* = 0.009) after 12-months in 27 rHTN patients with moderate-to-severe CKD. The efficacy of RDN to preserve renal function was evidenced by retrospective analyses showing patients experienced an average decline in eGFR of −4.8 ± 3.8 ml/min/1.73 m^2^ per year prior to the RDN procedure. Interestingly, the magnitude of change in 24-h ambulatory systolic BP at 12-months was not shown to predict change in eGFR. This contrasts with Kiuchi et al.'s study (Kiuchi et al., 2014) and suggests RDN may attenuate renal function decline via mechanisms unrelated to BP. Importantly, neither procedural complications nor evidence of acute kidney injury following RDN was reported in patients.

Central sympathetic activity present in ESRD patients is driven principally by afferent renal nerve signaling from the diseased native kidneys (Converse et al., 1992). To date, the use of RDN to reduce increased cardiovascular mortality in ESRD patients with renal hypertension has been explored in a limited number of clinical studies (Schlaich et al., 2009b; Spinelli et al., 2014). In a proof-of-concept study of 9 patients with ESRD and hypertension, RDN resulted in sustained reductions in office SBP of −18, −16, and −28 mmHg at 3−, 6−, and 12-months, respectively (Schlaich et al., 2009b). Two patients (*n* = 2) who underwent additional measures of sympathetic activity at 3-months, also displayed reductions in muscle sympathetic nerve activity, and renal and whole body noradrenaline release. With respect to safety, only 2 patients developed perioperative femoral pseudo-aneurysms that were resolved without further sequelae. Findings from the pilot trial are supported by an elegant case series involving four patients (age range: 22–65 years) with ESRD and difficult anatomy (renal arteries < 4 mm) (Spinelli et al., 2014), which reported a mean reduction in 24-h ambulatory BP of −36/−16 mmHg at 12-months follow-up. With the exception of notches detected on the final angiogram, no other procedure related complications were reported. Larger, randomized controlled clinical trials are planned or currently ongoing to substantiate the seminal findings that RDN is a safe and efficacious therapeutic approach to both lower BP and regulate sympathetic activity in patients with impaired kidney function.

### Chronic heart failure

Chronic heart failure (CHF) patients often exhibit renal dysfunction with augmented sympathetic tone (Hasking et al., 1986). Renal and cardiac noradrenaline spillover is a stronger predictor of mortality (Hasking et al., 1986; Petersson et al., 2005) than whole-body noradrenaline spillover in CHF patients suggesting chronic renal afferent nerve signaling is involved in the maintenance and progression of the pathological state.

Two small-scale trials assessing the potential benefits of RDN have been undertaken in CHF patients (Davies et al., 2013). The REACH pilot study evaluated the safety of RDN in 7 normotensive patients with CHF (class III–IV) and mean ejection fraction of 45% (Davies et al., 2013). At 6-months, all patients showed an improvement in their functional capacity (as assessed by a 6-min walk test) and overall quality of life. Importantly, no procedural complications or symptomatic adverse effects were reported and renal hemodynamics and function were also preserved.

In a larger observational study (OLOMOUC 1 Study) involving 51 patients with advanced CHF (mean ejection fraction 25%), RDN was associated with improved ventricular systolic function compared to standardized drug therapy (beta-blockers, ACE inhibitors or ARBs and diuretics) (Taborsky et al., 2012). Of the 26 patients randomized to RDN, left ventricular ejection fraction improved by an average of 6% at 12-months follow-up (mean ± SD: 25 ± 12% vs. 31 ± 14%; *p* < 0.01). No change was reported in the 25 patients randomized to standardized drug therapy (*p* = 0.36). Patients in the RDN group also reported significant reductions in heart rate and NT-pro brain natriuretic peptide levels and twice as fewer hospitalisations (31 vs. 72%; *p* < 0.001) during follow-up. Both treatments were shown to preserved patients renal function (as measured by eGFR; *p* = 0.55).

The stabilizing effect on RDN on heart failure progression is currently being investigated in several larger, randomized controlled trials. Studies in diastolic heart failure patients are also ongoing. Specifically, the DIASTOLE study will use magnetic resonance imaging to assess the efficacy of RDN to improve diastolic functional parameters in a multicenter-randomized controlled trial of 60 heart failure patients with preserved ejection fraction (Verloop et al., 2013).

### Left ventricular hypertrophy

Left ventricular hypertrophy (LVH) is an indicator of end-organ damage in arterial hypertension and is associated with increased cardiovascular morbidity and mortality risk, independent of BP (Ruilope and Schmieder, 2008; Bombelli et al., 2009). Chronic sympathetic nerve activity is shown to mediate hypertension-induced cardiac remodeling (Perlini et al., 2005). In hypertensive adults, cardiac noradrenaline spillover is positively related to LV mass index, suggesting a direct relationship between increased cardiac sympathetic activity in the development of LVH (Schlaich et al., 2003).

Two echocardiographic pilot studies have reported beneficial effects of RDN on measures of cardiac function and structure (Brandt et al., 2012; Schirmer et al., 2014). In 46 patients with rHTN, RDN was shown to significantly reduce LV mass (by 17%) and interventricular septum thickness (by −1.6 mm) at 6-months follow-up (both *p* < 0.001). Improvements in LV ejection fraction, LV end-systolic volume, diastolic LV filling pressures and isovolumic relation time were also observed (all *p* < 0.006). With the exception of LV mass and left atrial size, which was higher at 6-months follow-up compared to RDN patients, no other significant changes were observed in the control patients (*n* = 18). The effect of RDN on LV mass regression and diastolic function, while most prominent in patients who displayed the greatest reduction in systolic BP, was not exclusively associated with 6-month BP changes, suggesting RDN exerts effects on cardiac remodeling independent of BP. Schirmer et al. similarly reported in 66 overweight patients with rHTN a reduction in LV mass, improved diastolic function and increased vascular compliance at 6-months following RDN (all *P* < 0.001) (Schirmer et al., 2014). Compared to baseline, reductions in office BP (mean reduction −22/−10 mmHg) and heart rate (mean reduction −7 bpm) were also observed (both *p* < 0.001). With the exception of vascular compliance, which was directly correlated with BP reduction (*r*^2^ = 0.29, *p* < 0.001), the degree of LV mass regression or diastolic improvement reported post-RDN was not dependent on the magnitude of reduction in BP or heart rate.

A recent prospective multi-center, blinded study using cardiac magnetic resonance imaging (a more reliable measure of cardiac function and morphology), confirms the aforementioned echocardiographic findings that RDN reduces LV mass regression and improves LV ejection fraction largely independent of the significant changes in BP (Mahfoud et al., 2014). Compared to 17 patients who received medical treatment only, 55 patients with rHTN treated with RDN reported a significant 7.1% reduction in LV mass (mean ± SD: 46.3 ± 13.6 vs. 43.0 ± 12.6 g/m^1.7^; *P* < 0.001) and 3.4% improvement in ejection fraction (mean ± SD: 55.7 ± 11.1 vs. 57.6 ± 9.3%; *P* = 0.048) at 6-months follow-up. Importantly, LV mass was reduced in those patients who did not show significant clinical reductions in systolic BP at 6-months (defined as systolic BP reduction < 10 mmHg). In a sub-group of patients (*n* = 19) with markedly reduced systolic LV ejection fraction (defined as <50%) at baseline, the effect of RDN was even more pronounced resulting in a 7.3% improvement in LV ejection fraction; *P* < 0.001) at 6-months. Left ventricular wall stress, defined as a function of chamber size and configuration, thickness of the ventricular wall and intraventricular pressure was also significantly reduced following RDN at 6-months (*p* = 0.03). No changes in any cardiac parameters were reported in the control group.

Given the current findings support RDN having a prognostic benefit on LVH regression in resistant hypertensive patients who are at heighten risk for heart failure, larger studies analyzing clinical outcomes are warranted.

### Arrhythmias

Preliminary evidence suggests that RDN may have a salutary effect in the management of recurrent arrhythmias in heart failure patients, particularly those with atrial fibrillation (Pokushalov et al., 2012) and ventricular tachycardia (Ukena et al., 2012; Hoffmann et al., 2013), via a reduction in BP and central sympathetic cardiac stimulation.

The potential antiarrhythmic efficacy of RDN was first described in a normotensive porcine model (Linz et al., 2013a) and canine with pacing-induced heart failure model (Zhao et al., 2013). First-in-man experience comes from a small prospective study of 27 patients with refractory symptomatic atrial fibrillation and rHTN (Pokushalov et al., 2012). Compared to patients who underwent usual treatment for atrial fibrillation pulmonary vein isolation (PVI) (*n* = 14), patients who underwent a combined therapy of PVI and RDN (*n* = 13) exhibited a two-fold reduction in the occurrence of atrial fibrillation episodes (defined as <30 s of atrial fibrillation during 9-months follow-up). Patients on combined therapy also demonstrated a significant and sustained BP reduction of −25/−10 mmHg and reduction in LV mass of approximately 10% at follow up. Findings from a two-study meta-analysis suggest the salutary effects of RDN when used as an adjunct therapy to PVI on atrial fibrillation reoccurrence is even more pronounced in patients with severe rHTN (Pokushalov et al., 2014). A recent study by McLellan et al. suggests electrical remodeling, specifically an increase in global and atrial conduction velocity, may in conjunction with structural changes promote a reduction in atrial fibrillation reoccurrence following RDN (McLellan et al., 2015).

Ventricular tachyarrhthmias (VTA) are associated with a high, irregular heart rate (>100 bpm) and significant risk for sudden death. Elevated cardiac sympathetic nerve activity has been linked to the pathogenesis of VTAs (Meredith et al., 1991) with structural changes in myocardial tissue (i.e., myocardial hypertrophy and heart failure) caused by elevations in BP thought to predispose patients to VTA (Anderson, 1984; Bryant et al., 1999).

In a porcine model of acute coronary myocardial infarction, RDN was shown to significantly reduce the incidence of ventricular arrhythmia compared to a sham procedure (86 vs. 17%; *p* = 0.03) (Linz et al., 2013b). The efficacy of RDN to suppress ventricular tachycardia in adults has only been explored in a few case studies involving patients with ventricular electrical storm (Ukena et al., 2012; Hoffmann et al., 2013). Ukena et al. reported the first-in-man experience in 2 patients suffering from asymptomatic CHF (non-obstructive hypertrophic and dilated cardiomyopathy, NYHA III) and treatment resistance ventricular electrical storm (Ukena et al., 2012). In both patients, RDN was shown to eliminate the occurrence of ventricular tachyarrhythmic episodes at 6-months without altering BP. In a 63 year old male with recurrent ventricular electrical storm in the setting of acute myocardial infarction, the use of RDN in combination with ventricular tachycardia catheter ablation (standard therapy) was shown to eliminate both ventricular tachycardia and ventricular fibrillation episodes at 23, 100, and 160 days follow-up (Hoffmann et al., 2013). The patient also experienced normalization of their BP, which warranted a reduction in their antihypertensive medication.

While the observations reported in these case studies are promising for RDN as an adjunctive therapy for patients with serious cardiac electrical instability they underscore the need for future randomized controlled trials.

### Metabolic diseases

Accumulating data from animal and human studies suggest chronic sympathoexcitaion plays a pivotal role in the etiology and complications of metabolic conditions. Even in the absence of hypertension, elevated urinary noradrenaline levels, increased efferent muscle sympathetic nerve activity, and elevated rates of plasma noradrenaline spillover, are present in patients with obesity, insulin resistance and the metabolic syndrome (Lee et al., 2001; Grassi et al., 2005; Straznicky et al., 2008; Schlaich et al., 2015).

Retrospective sub-cohort analyses of 37 rHTN patients who underwent RDN in the Symplicity HTN-1 Study, showed improvements in office BP (−32/−12 mmHg; *P* < 0.001) were accompanied by significant reductions in fasting plasma glucose, insulin, C-peptide and 2-h post load glucose levels at 3-months follow-up (all *p* > 0.04) (Mahfoud et al., 2011). Insulin sensitivity as measured by the HOMA-IR and IS_QUICKI_ index measures was also improved by −62 and 13%, respectively, at 3-months follow-up (both *P* = 0.001). Importantly, these beneficial metabolic alterations were preserved in patients diagnosed with type 2 diabetes at baseline (*n* = 20). No BP or metabolic improvements were reported in the control group (*n* = 13) who continued their usual medication regime.

These impressive findings contrast with the recently published Denervation of the Renal Artery in Metabolic Syndrome (DREAMs) study that prospectively reported no effect of RDN on measures of insulin sensitivity after 12-months in 29 patients with metabolic syndrome on ≤1 antihypertensive and/or diabetic medication (Verloop et al., 2015). Of note, whole-body sympathetic activity, as measured by microneurography (*n* = 10), and heart rate variability (*n* = 26) did not change at 12-months post-RDN despite a modest reduction in 24-h ambulatory BP (mean change from baseline −6 ± 12/−5 ± 7 mmHg; *p* < 0.02).

Improved glucose metabolism following RDN has been reported in patients with polycystic ovary syndrome (Schlaich et al., 2011) and obstructive sleep apnea (Witkowski et al., 2011), two conditions that are characterized by multiple metabolic disturbances. In 2 obese women with polycystic ovary syndrome, RDN was shown to lower fasting plasma glucose, improve insulin sensitivity (assessed by euglycaemic hyperinsulinemic clamp) and reduce both muscle sympathetic nerve activity and whole-body noradrenaline spillover at 3-months follow-up (Schlaich et al., 2011). Importantly, these metabolic effects were shown to occur in the absence of any changes in body weight. In 10 patients with obstructive sleep apnea, RDN was associated with improved 2-h glucose levels (median: 7.0 vs. 6.4 mmol/L; *p* = 0.05) during an oral glucose tolerance test and reduced HbA1c levels (median: 6.1% vs. 5.6%; *p* < 0.05) at 6-months follow-up (Witkowski et al., 2011). Improvements in office BP (−34/−13 mmHg: *p* < 0.01) and severity of obstructive sleep apnoea for 80% of patients was also observed. Overall, the evidence suggests acute improvements in insulin resistance and glycemic control ensue from RDN. Understanding the longer term effects of RDN in patients with metabolic disease is expected to be a focus for further investigation.

## Future of renal denervation: where to from here?

Despite the recent disappointment of Symplicity HTN-3, support remains for the efficacy of RDN in the real world setting (Bohm et al., 2015). Recent analyses from Medtronic's Global SYMPLICITY Registry showed 998 patients who underwent RDN experienced a significant lowering in office and ambulatory systolic BP by an average of −19.8 and −9.2 mmHg, respectively, after 6-months. For patients with a baseline systolic BP ≥ 160 mmHg, the BP reduction following RDN was even more pronounced. The registry also confirmed the well-established short-term safety profile of RDN with only six procedure-related events reported (<1% of the cohort) during the 6-months. Collectively, the results lend support to RDN being a safe and viable therapeutic option in patients with severe rHTN when traditional pharmacotherapy has failed.

In 2014, the Joint UK societies' consensus statement on RDN for rHTN called for a temporary moratorium on RDN in routine clinical practice following the Symplicity HTN-3 announcement but was hesitant to abandon the therapy entirely, citing the need for further research (Lobo et al., 2015). In this respect, Symplicity HTN-3 has been invaluable in helping guide the design and execution of future clinical studies. The finding that patients who received ablations in all four quadrants of the renal artery were more likely to experience significant reductions in BP in Symplicity HTN-3 (Bhatt et al., 2014) coupled with contemporary anatomical insights into the distribution of renal artery nerves (Sakakura et al., 2014) demonstrates certain ablation patterns are more efficacious for lowing BP than others. Indeed, there is now a preferential shift toward the use of modalities that circumferentially ablate nerves toward the distal portion of both renal arteries. Patient selection is also critical with meticulous identification of true treatment-rHTN using ambulatory BP monitoring during a period of stable medication (>8 weeks) confirmed by drug adherence testing necessary to eliminate patients without “neurogenic” hypertension and those who simply are non-compliant with their BP medication.

At present, there is no intraprocedural marker to confirm whether RDN has been successfully achieved. Instead, physicians have naively relied on changes in BP to define procedural success. Noradrenaline spillover testing has been performed in small sub-groups of patients to quantify whole-body (Krum et al., 2009) and renal sympathetic nerve activity (Esler, 2014a) prior to and 30-days post RDN. While a reduction in renal noradrenaline spillover correlates with a reduction in BP, the response can be highly variable between individual patients (Esler, 2014b). Furthermore, the validated test is not suitable for use in large-scale trials.

Identification of a novel biomarker of renal nerve injury that can be measured in either urine or plasma immediately following RDN would be the ne plus ultra to inform interventionists of procedural success. Recent animal data suggests intraluminal electrical stimulation of renal arteries pre- and post-RDN may provide valuable insight in the acute efficacy of the procedure. Indeed, Chinushi et al. reported an increase in BP, serum catecholamines and heart rate variability immediately following high-frequency electrical stimulation of renal arteries in a hypertensive canine model, which was attenuated following RDN (Chinushi et al., 2013). Adenosine infusion into the renal artery, which under normal conditions potentiates a rise in BP, has also been suggested as an immediate measure of renal afferent nerve ablation success following RDN (Esler, 2014a).

## Conclusion

Latest clinical trial data suggests RDN is a safe treatment option in patients with true rHTN that is most efficacious when used as an adjunct therapy to intensive pharmacotherapy. It is hoped the development of next generation ablation modalities that enable circumferential ablation coupled with more stringent screening of true treatment rHTN will help improve the clinical efficacy of RDN. Encouraging data from a number of pre-clinical studies highlights that clinical benefits beyond BP reduction can be gained following RDN in patients with renal disease, heart failure, arrhythmias and metabolic-related disease. However, caution is warranted not to over-interpret the findings of these small studies with larger, randomized controlled trials needed before the application of RDN becomes routine in clinical practice for these patient cohorts.

## Author contributions

AT and MS contributed to the conception and interpretation of data in the review; AT prepared the manuscript, tables, and figures; MS reviewed and edited the manuscript; AT and MS approved the final version of the manuscript and are accountable for all aspects of the review presented.

## Funding

MS is supported by a Senior Research Fellowship from the National Health and Medical Research Council (NHMRC) of Australia. AT is supported by an NHMRC of Australia Grant. This research was funded in part by the grants from the National Health and Research Council of Australia (NHMRC) and the Victorian Government's Operational Infrastructure Support Program. MS is an investigator in studies sponsored by Medtronic and has received honoraria and lecture fees.

### Conflict of interest statement

MS is an investigator in studies sponsored by Medtronic and has received honoraria and lecture fees. AT has no conflicts of interest to disclose.
